# Mitochondria-related miR-574 reduces sperm ATP by targeting *ND5* in aging males

**DOI:** 10.18632/aging.103141

**Published:** 2020-05-07

**Authors:** Jinzhao Ma, Qiwei Chen, Shuxian Wang, Rujun Ma, Jun Jing, Yang Yang, Yuming Feng, Zhichuan Zou, Yu Zhang, Xie Ge, Tongmin Xue, Kuan Liang, Siyuan Cao, Dandan Wang, Li Chen, Bing Yao

**Affiliations:** 1 Center of Reproductive Medicine, Nanjing Jinling Hospital, The First School of Clinical Medicine, Southern Medical University, Nanjing 210002, Jiangsu, China; 2 Center of Reproductive Medicine, Nanjing Jinling Hospital, Clinical School of Medical College, Nanjing University, Nanjing 210002, Jiangsu, China; 3 Institute of Laboratory Medicine, Nanjing Jinling Hospital, Clinical School of Medical College, Nanjing University, Nanjing 210002, Jiangsu, China; 4 Center of Reproductive Medicine, Nanjing Jinling Hospital, School of Medicine, Jiangsu University, Zhenjiang 212002, Jiangsu, China; 5 Jinling Hospital Department Reproductive Medical Center, Nanjing Medicine University, Nanjing 210002, Jiangsu, China; 6 School of Life Science, Nanjing Normal University, Nanjing 210002, Jiangsu, China

**Keywords:** male aging, sperm, miRNA, mitochondria, mt-ND5

## Abstract

Couples are delaying childbearing in recent decades. While women experience a notable decrease in oocyte production in their late thirties, the effect of advanced paternal age on reproduction is incompletely understood. Herein, we observed that numerous miRNAs, including miR-574, increased in the sperm of aging males, as indicated by high-throughput sequencing. We demonstrated that miR-574 was upregulated in the sperm of two aging mouse models and was related to inferior sperm motility as an adverse predictor. Moreover, we proved that miR-574 suppressed mitochondrial function and reduced cellular ATP production in GC2 cells. Mechanistically, we demonstrated that miR-574 regulated mitochondrial function by directly targeting mt-ND5. Our study revealed an important role of miR-574 in sperm function in aging males and provided a fresh view to comprehend the aging process in sperm.

## INTRODUCTION

An increasing number of men are fathering children at an older age than in the past. Due to societal pressures, increased life expectancy, assisted reproduction techniques (ART) and the use of modern contraception, the average reproductive age of couples has risen visibly in recent decades, with the mean age of their first reproduction in mid- or late-thirties in many countries [1]. In contrast to female reproductive physiology, male functions do not stop at a defined time such as menopause, and spermatogenesis continues throughout life. Although increasing maternal age is well established as a negative indicator of fertility, reproductive success and offspring fitness, the influence of paternal age on reproduction is incompletely understood.

Multiple epidemiologic studies have been conducted to examine the relationship between paternal age and semen quality, and numerous studies have reported age-related declines in semen quality, including semen volume, sperm motility and sperm morphology [2–4]. More recently, a systematic review using data from 90 studies (93,839 subjects) indicated that semen volume, percentage motility, progressive motility and normal morphology declined with age, while DNA fragmentation increased with age [5]. These studies suggest that advanced paternal age tends to be associated with a decline in semen quality [5, 6].

The mechanisms responsible for age-dependent patterns of decline in semen traits are not fully understood, but the damage from reactive oxygen species (ROS) is thought to be an important contributor [5]. ROS are produced in the mitochondria and their abnormal increase usually indicates mitochondrial dysfunction. Increased ROS were correlated with decreased sperm motility and accumulated DNA fragmentation at both the nuclear and mitochondrial levels, which in turn exacerbated the sperm dysfunction and abnormalities [7]. Sperm mitochondria are involved in many essential processes in reproduction, such as sperm motility, hyperactivation, capacitation and acrosome reaction. Mitochondrial function regulation could be an instrumental strategy to modulate the sperm function, including sperm motility.

In addition to possible mutagenic events, aging is associated with widespread epigenetic changes, and epigenetic alterations in sperm are increasingly implicated in beneficial or deleterious effects on the sperm function, and embryo or offspring development [8, 9]. MicroRNA (miRNA) in sperm could be a typical mediator of epigenetic regulation and may participate in the modulation of sperm function. Recently, several studies have demonstrated that miRNAs, encoded by the nuclear genome or mitochondrial genome, not only regulate nuclear genome encoding mitochondria-related proteins, but also could translocate into the mitochondria and regulate mitochondrial genome expression [10]. Zeng and colleagues demonstrated that miRNAs could induce mitochondrial dysfunction, increase ROS production, activate inflammasomes and pyroptosis, and promote the process of atherosclerosis [11]. Ran and colleagues observed that miR-151a-5p decreased mitochondrial respiratory activity and adenosine triphosphate (ATP) levels by targeting the mitochondrial transcription mt-Cytb in asthenozoospermia [12]. These results suggest that miRNAs might modulate sperm function through a mitochondria-dependent pathway.

In order to explore the expression patterns of advanced age on reproduction, our group previously performed high-throughput sequencing of small RNAs in sperm, oocytes and embryos of aging and young mice. A mass of miRNAs differentially expressed in sperm, oocytes and embryos in aging and young mice were observed. Excluding differentially expressed miRNAs in oocytes, we found that 101 miRNAs were specifically differentially expressed in the sperm of the aging and the young mice. We considered that some of the miRNAs may participate in the modulation of sperm function.

It is widely believed that older males are susceptible to accumulating harmful mutations and compounds. Numerous studies have identified paternal age as a risk factor for spontaneous abortion and may contribute to adverse reproductive outcomes, such as schizophrenia, autism and several X-linked recessive and autosomal dominant disorders [13]. Moreover, lots of studies have demonstrated that sperm-borne miRNAs are involved in embryo development [14]. Microinjection of miRNAs into pronuclei (PN) stage embryos provides direct evidence that alterations of miRNA abundance in early embryo development could even induce phenotypes in adult offspring, including cardiac hypertrophy, overgrowth, obesity and behavioral defects [15–19]. The microinjection of miRNAs dysregulated in sperm by a father’s chronic stress might cause targeted degradation of stored maternal mRNAs and induce a cascade of molecular events that ultimately perturb early embryo development and transmit phenotypes to the offspring [20]. We wondered that whether the differentially expressed miRNAs in sperm contribute to early embryo development. To this end, we overlapped the specific miRNAs in sperm with the differentially expressed miRNAs in the embryo, and identified 33 miRNAs that might contribute to embryo development from the sperm of aging males.

By comparison with the validated miRNAs enriched in mitochondria from different cell lines and tissues, we found that miR-574 was the miRNA that might be associated with mitochondria. MiR-574 is highly conserved between humans and mice and has been reported to be associated with testis development and reproduction [21]. Pan and colleagues have demonstrated that miR-574 negatively impacts oocyte quality and meiotic progression by targeting HAS2 during in vitro maturation (IVM) [22]. We therefore hypothesize that mitochondria-related miR-574 may play a role in sperm function or even early embryo development.

In the present study, we sought to investigate the role of miR-574 in sperm function and early embryo development, and address the mechanisms by which miR-574 participates in the process of semen quality decrease induced by male aging. We found that miR-574 was upregulated in the sperm of aging males and was related to poor sperm motility. Moreover, we proved that miR-574 suppressed the mitochondrial function and reduced cellular ATP production by directly targeting mt-ND5. However, we only observed a downward trend of miR-574 on embryonic development. Overall, we propose the important roles of miR-574 in the sperm function of aging males.

## RESULTS

### Aberrant expression of miRNAs in the sperm of aging males

**Figure 1:**
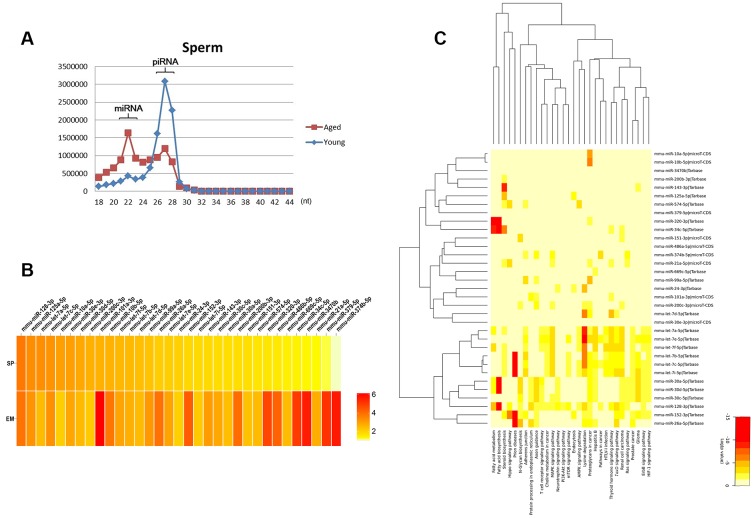
**Aberrant expression of miRNAs in the sperm of aging males.** (**A**) The distribution of miRNAs and piRNAs in the sperm of the aged and the young males. (**B**) The 33 miRNAs co-upregulated in the sperm and embryos of the aged compared to the young group. (**C**) The potential pathways of the 33 miRNAs predicted by the online bioinformatics tool mirPath.

In order to explore the expression patterns of advanced age on reproduction, our group previously performed high-throughput sequencing of small RNAs in sperm, oocytes and embryos of aged and young mice (data not shown). Numerous microRNAs (miRNAs) or piwi-interacting RNAs (piRNAs) were differentially expressed between the aged and the young. Further analysis of sperm data revealed that the expression patterns of sperm from aged and young mice were markedly different. The distribution of miRNAs was higher in the aged group, while the distribution of piRNAs was higher in the young group (Figure 1A). As piRNAs were expressed mainly in pachytene spermatocytes and spermatids in the testes of mammals [23], we focused on the differentially expressed miRNAs between the aged and young groups. We found 162 miRNAs that were differentially expressed, of which 160 were upregulated in the sperm of aged males. After excluded differentially expressed miRNAs in oocytes, we found 101 miRNAs that were specifically differentially expressed in sperm. As sperm miRNAs might participate in embryo development, we overlapped the specific miRNAs in sperm with the differentially expressed miRNAs in embryos, and obtained 33 miRNAs that might contain the contributor of embryo development from the sperm of aging males (Figure 1B). By using the online bioinformatics tool mirPath v.3 [24], we found that these miRNAs were mainly involved in pathways including fatty acid metabolism, protein processing, mTOR signaling, Hippo signaling and steroid biosynthesis (Figure 1C). Collectively, these results indicated that numerous microRNAs were differentially expressed in the sperm of aging males and might be involved in embryo development.

### Mitochondria-related miR-574 was upregulated in the sperm of aging males and was related to poor sperm motility

As numerous studies have suggested that advanced paternal age tends to be associated with a decline in semen quality including sperm motility, and mitochondria-related miRNAs have been found to be associated with multiple diseases, including asthenozoospermia, by regulating mitochondrial functions, we wondered whether the differentially expressed miRNAs from sperm of the aged and young groups could contain a number of mitochondria-related miRNAs that regulate mitochondrial function to alter sperm motility similar to their function in asthenozoospermia. By comparison of the validated miRNAs enriched in mitochondria from different cell lines and tissues [25, 26], we found that miR-128, miR-125a, let-7b, miR-24 and miR-574 were the miRNAs that might be associated with mitochondria. Moreover, we found that miR-574 was highly conserved between humans and mice (Supplementary Figure 1A) and has been reported to be associated with testis development and reproduction [21]. We then chose miR-574 for further study.

**Figure 2:**
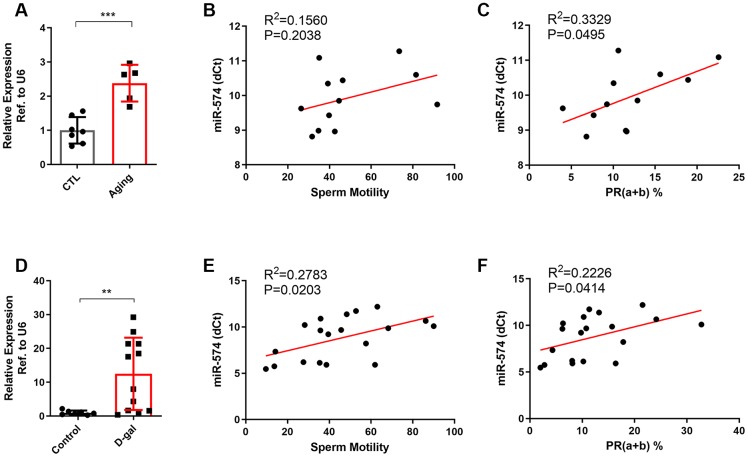
**miR-574 was upregulated in the sperm of aging males and was related to poor sperm motility.** (**A**) The expression of miR-574 in the sperm of the natural aging model. *t* test was used to compare the differences between the two groups. ***p<0.001. (**B**, **C**) Scatter plot of sperm motility, progressive motility and the miR-574 expression in the sperm of the natural aging model. (**D**) The expression of miR-574 in the sperm of the D-gal-induced aging model. **p<0.01. (**E**, **F**) Scatter plot of sperm motility, progressive motility and the miR-574 expression in the sperm of the D-gal-induced aging model.

To confirm the small RNA-seq results, we established two aging mouse models. In the natural aging model, the older-age mice presented characteristics of aging, such as thinning of hair, and hypoactivity (Supplementary Figure 1B). The body weight of aging mice was higher than that of young mice, but no significant difference in testicular weight was found between the two groups, leading to an obvious decrease of testicular organ index in the aging group (Supplementary Figure 1C–1E). We then analyzed the sperm parameters of the two groups by computer-assisted sperm analysis (CASA) and found a significant decrease in sperm concentration, total motility and progressive motility (PR) in the aging group (Supplementary Figure 1F–1H). We also observed a similar decline in serum testosterone in the aging group (Supplementary Figure 1I). By using hematoxylin and eosin (H&E) staining and electron microscopy, we found that the aging mice exhibited more vacuoles in the seminiferous tubules and more malformed mitochondria in the testes (Supplementary Figure 1J–1K). These data suggested that the aging group mice presented an evident aging phenotype. We then detected the expression of miR-574 in the sperm of the two groups and found that miR-574 was significantly upregulated in the aging group (Figure 2A). Further analysis of the relationship between sperm parameters and the miR-574 expression revealed that miR-574 expression was inversely related to sperm motility, especially to progressive motility (Figure 2B, 2C), but not to sperm concentration (Supplementary Figure 1L).

We then established a D-gal-induced aging mouse model by injecting D-gal subcutaneously into the mice daily for 42 days and found that the D-gal treated mice presented few characteristics of aging in appearance (Supplementary Figure 2A). No significant differences in body weight, testicular weight or testicular organ index were found between the D-gal-treated mice and the control mice (Supplementary Figure 2B–2D). Subsequently, we analyzed the sperm parameters of the two groups by CASA and found a significant decrease in sperm concentration, total motility and PR in the D-gal-treated group (Supplementary Figure 2E–2G), which was consistent with our expectations. We also observed a similar decline in serum testosterone in the D-gal-treated group (Supplementary Figure 2H). By using H&E staining and electron microscopy, we found that the D-gal-treated mice exhibited more vacuoles in the seminiferous tubules and more malformed mitochondria in the testes, similar to the natural aging models (Supplementary Figure 2I, 2J), suggesting that the D-gal-treated mice presented an analogous phenotype of aging. We then detected the expression of miR-574 in the sperm of the two groups and found that miR-574 was significantly upregulated in the D-gal-treated group (Figure 2D). Further analysis of the relationship between sperm parameters and the miR-574 expression revealed that miR-574 expression was inversely related to sperm concentration, sperm total motility and progressive motility (Supplementary Figure 2K and Figure 2E, 2F).

Moreover, we collected clinical semen samples from the Reproductive Medicine Center of Nanjing Jinling Hospital and detected the expression of miR-574 in the sperm of patients more than or less than 40 years old. However, we only observed a trend that miR-574 was seemingly upregulated in the sperm of patients more than 40 years old (Supplementary Figure 2L). It was considered that confounding factors other than age were brought in the detection and the patient fertility status might be variable and different from that of the laboratory animals. Collectively, these experiments indicated that mitochondria-related miR-574 was upregulated in the sperm of aging males and was related to poor sperm motility.

### MiR-574 impaired mitochondrial function and reduced cellular ATP production

**Figure 3:**
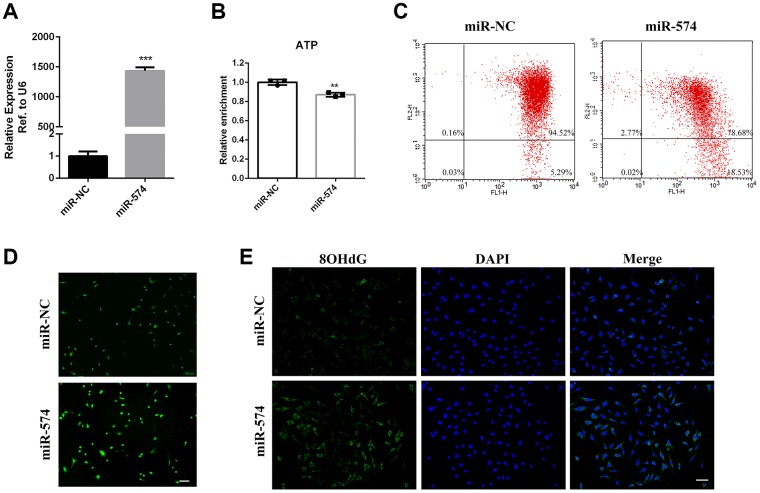
**Overexpression of miR-574 impaired mitochondrial function and reduced cellular ATP production.** (**A**) MiR-574 overexpression efficiency detection in GC2 cells transfected with miR-574 mimic. (**B**) Overexpression of miR-574 decreased intracellular ATP levels. (**C**) The mitochondrial membrane potential of the miR-574 overexpression group was significantly inhibited compared with control groups, as assayed by flow cytometry. (**D**) MiR-574 increased the intracellular ROS levels in GC2 cells. Scale bar=100 μm. (**E**) Immunofluorescence was used to detect intracellular 8-OHdG levels. MiR-574 significantly increased intracellular 8-OHdG (green) levels compared with the control group. The nuclei were stained blue with 4,6-diamidino-2-phenylindole (DAPI). Scale bar=100 μm.

To identify the potential function of miR-574, we overexpressed miR-574 in GC2 cells by transfection of miR-574 mimics (Figure 3A). Then, we measured GC2 cellular ATP levels and found that miR-574 could decrease ATP production in GC2 cells (Figure 3B). Furthermore, we examined the effect of miR-574 on mitochondrial membrane potential (MMP). Flow cytometry results showed that miR-574 increased the ratio of Q4 district/ Q2 district compared with that in the control group (Figure 3C), indicating that miR-574 might cause mitochondrial membrane potential abnormalities. Moreover, ROS and DNA damage levels (marked by 8-OHdG) were detected in GC2 cells transfected with miR-574. Our results demonstrated that miR-574 significantly increased cellular ROS and DNA damage levels (Figure 3D–3E). Together, these results indicated that miR-574 could impair mitochondrial function and reduce cellular ATP production.

### MiR-574 depletion relieved mitochondrial dysfunction and increased cellular ATP production

**Figure 4:**
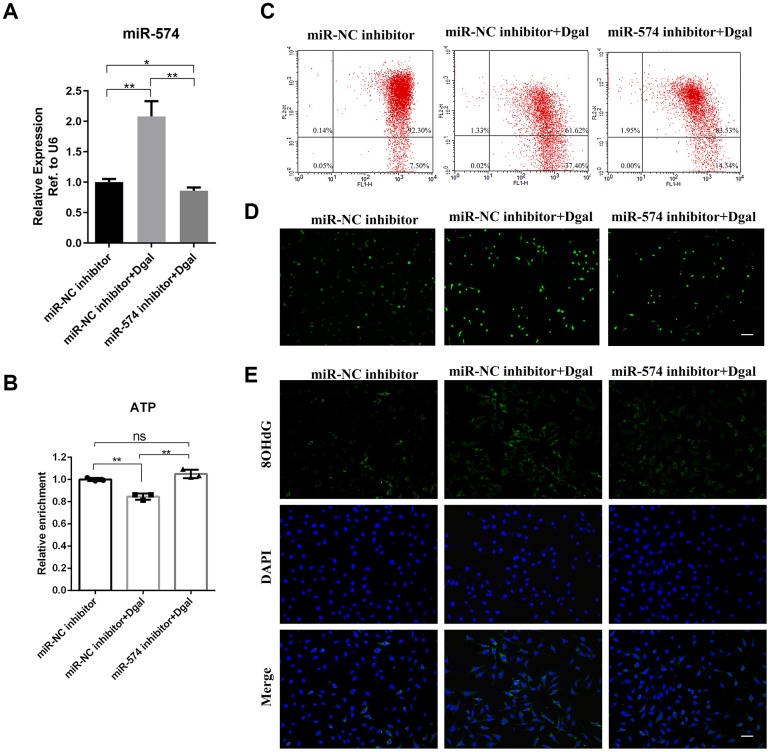
**MiR-574 depletion relieved mitochondrial dysfunction and increased cellular ATP production.** (**A**) MiR-574 was upregulated in GC2 cells by treatment with D-gal (50 mmol/L) and reduced after miR-574 inhibitor addition. (**B**) D-gal significantly inhibited the ATP levels, and miR-574 inhibitor alleviated D-gal induced ATP decrease. (**C**) The mitochondrial membrane potential of the D-gal group was significantly inhibited compared with control groups, and miR-574 inhibitor mitigated the D-gal induced mitochondrial membrane potential abnormalities, as assayed by flow cytometry. (**D**, **E**) D-gal significantly increase the cellular ROS and 8-OHdG levels, and miR-574 inhibitor relieved this increase. The nuclei were stained with 4,6-diamidino-2-phenylindole (DAPI). Scale bar=100 μm.

We treated GC2 cells with D-gal and found that D-gal increased the expression of miR-574. To further explore the role of miR-574, we reduced the expression of miR-574 in GC2 cells by transfection with a miR-574 inhibitor (Figure 4A). Then, we measured the cellular ATP levels and found that D-gal decreased the ATP production in GC2 cells and that the miR-574 inhibitor alleviated this decrease (Figure 4B). Furthermore, flow cytometry results showed that D-gal increased the ratio of Q4 district/ Q2 district and that the miR-574 inhibitor mitigated this effect (Figure 4C). Moreover, ROS and DNA damage levels were detected in GC2 cells treated with D-gal or miR-574 inhibitor. We observed that D-gal significantly increased cellular ROS and DNA damage levels, and the miR-574 inhibitor could relieve the increase (Figure 4D–4E). Overall, these results indicated that miR-574 depletion could relieve mitochondrial dysfunction and increase cellular ATP production.

### MiR-574 regulated mitochondrial function by directly targeting mt-ND5

**Figure 5:**
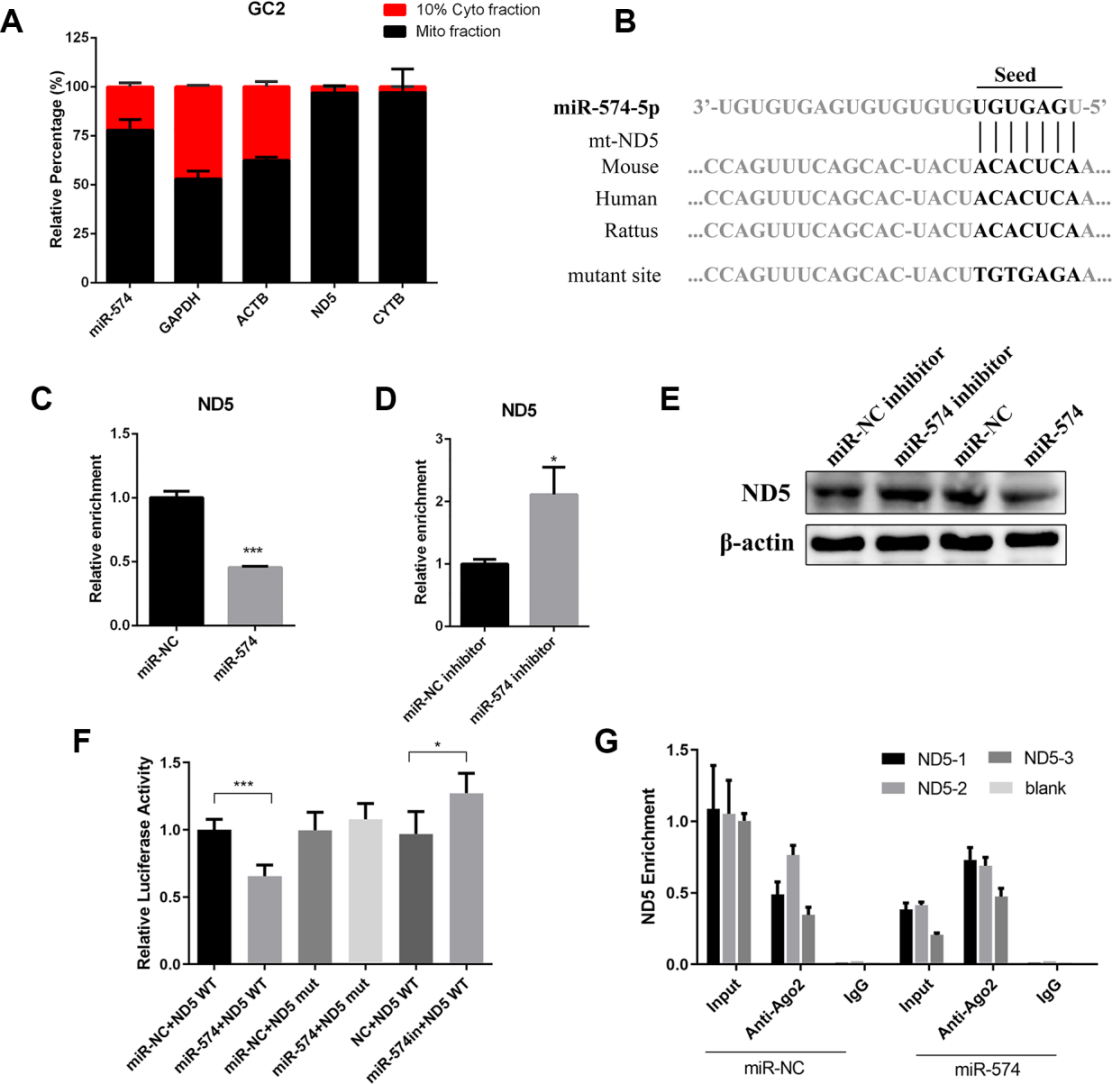
**MiR-574 regulated mitochondrial function by directly targeting mt-ND5.** (**A**) MiR-574 and positive control genes were detected in the mitochondrial fraction and cytoplasmic fraction in GC2 cells. (**B**) The putative site at which miR-574 binds to mt-ND5 in multiple species, the mutant vector was constructed by mutating miR-574 binding sites in mt-ND5. (**C**, **D**) mRNA levels of mt-ND5 in GC2 cells transfected with miR-574 mimic/inhibitor or their respective controls. ***p < 0.001, *p < 0.05. (**E**) Protein levels of mt-ND5 in GC2 cells transfected with miR-574 mimic/inhibitor or their respective controls. β-Actin was used to normalize the WB results. (**F**) Luciferase activity in GC2 cells co-transfected with miR-574 mimic/inhibitor or their respective controls and the WT/Mut luciferase reporter. (**G**) Anti-Ago2 RIP was performed in GC2 cells transiently overexpressing miR-574 or negative control, followed by qRT-PCR to detect mt-ND5 associated with Ago2.

Previous studies have demonstrated that many miRNAs and the core component of RISC, Ago2/Ago3, are found within the mitochondria and miRNAs may also translocate to the mitochondrial matrix to regulate mitochondrial gene expression [25]. We then isolated the mitochondrial fraction and cytoplasmic fraction and determined the miR-574 expression. We observed that miR-574 was expressed in the mitochondrial fraction, indicating that miR-574 may translocate to the mitochondria and regulate mitochondrial gene expression (Figure 5A). We predicted the target genes of miR-574 within the mitochondrial pathway by the bioinformatics tool RNA22 and found an evolutionarily conserved target site in the mt-ND5 gene (Figure 5B). We next detected the mt-ND5 expression in GC2 cells transfected with miR-574 mimics or inhibitor, and found that mt-ND5 mRNA and protein expression levels were significantly lower in cells transfected with miR-574 mimic and higher in cells transfected with miR-574 inhibitor than in cells transfected with negative control (Figure 5C–5E). To verify the relationship between miR-574 and mt-ND5, we constructed luciferase reporters containing either the wild-type (WT) or mutated (Mut) miR-574 binding sites (seed sequence) in mt-ND5 (Figure 5B). Overexpression of the miR-574 mimic reduced the luciferase activity of the WT reporter vector but not that of the mutated reporter vector (Figure 5F). It is known that miRNAs bind to their targets and cause translational repression and/or RNA degradation in an Ago2-dependent manner. To determine whether mt-ND5 is regulated by miR-574 in this manner, we conducted anti-Ago2 RIP in GC2 cells transiently overexpressing miR-574. The mt-ND5 pulldown by Ago2 was more highly enriched in miR-574-transfected cells, suggesting that miR-574 directly targets mt-ND5 (Figure 5G). Moreover, we observed that knockdown of mt-ND5 would relieve the increasing trend of cellular ATP production induced by the miR-574 inhibitor in D-gal-treated GC2 cells (Supplementary Figure 3A). Furthermore, we detected the mt-ND5 expression in the sperm of the two aging models, and found that mt-ND5 was significantly downregulated in the sperm of the aging group (Supplementary Figure 3B). Together, these results indicated that miR-574 regulated the mitochondrial function by directly targeting mt-ND5.

### Effects of miR-574 on early embryonic development

A previous study proved that miRNA might play a role in early embryonic development, and aging males exerted a certain level of fertility reduction and early embryonic dysplasia. However, the role of sperm miRNAs from aging males in early embryonic development is unclear. We then collected the zygotes and overexpressed miR-574 by microinjecting a miR-574 mimic into embryos to test the effect of miR-574 on early embryonic development. Our results indicated that the group injected with miR-574 showed a downward trend in embryonic development, but no statistical significance was observed (Supplementary Figure 4A). One possible explanation for this phenomenon might be that the function of a single miRNA is limited and the miRNA pools containing multiple miRNAs might be needed to observe a phenotypic effect in further investigations. We then detected the ROS of the embryos injected with miR-574 and found that miR-574 could obviously increase the ROS levels in embryos (Supplementary Figure 4B).

## DISCUSSION

In modern times, couples have begun to push the limits of conception to the point that children are commonly born to parents of advanced maternal and paternal age. Advanced maternal age has long been recognized as a risk factor for adverse reproductive outcomes. Advanced paternal age is also associated with increased reproductive risks, but the contributors and origins of differential risk for paternal aging remain poorly understood. Multiple effects of aging on sperm motility, sperm morphology and concentration indicate that the quality of spermatozoa declines over time, but few studies have shed light on the molecular mechanisms that hamper sperm function in older men.

Mitochondria participate in various biological processes, including energy production, calcium homeostasis and apoptosis, with their predominant roles differing among mammalian species. A decrease in sperm motility is a common phenomenon in aging males, and its etiology may be related to mitochondrial dysfunction due to its irreplaceable role in ATP generation. Accumulating evidence indicates that mitochondrial dysfunction may be associated with posttranscriptional regulation of gene expression by mitochondria-related miRNAs.

In this study, we found that numerous miRNAs increased in the sperm of aging males, and were involved in many pathways including fatty acid metabolism, protein processing, mTOR signaling, Hippo signaling and steroid biosynthesis (Figure 1A–1C). miR-574, a highly conserved miRNA reported to be associated with mitochondrial function, was selected for further study. We demonstrated that miR-574 was upregulated in the sperm of two aging mouse models and was related to inferior sperm motility as an adverse predictor (Figure 2A–2F). Moreover, we proved that miR-574 suppressed mitochondrial function, reduced cellular ATP production and increased cellular ROS and DNA damage levels in GC2 cells (Figures 3 and 4). Mechanistically, we demonstrated that miR-574 regulated mitochondrial function by directly targeting mt-ND5 (Figure 5). Furthermore, we evaluated the effects of miR-574 on early embryo development and found that microinjecting the miR-574 mimic into embryos resulted in a mild decreasing trend of embryonic development (Supplementary Figure 3).

Previous studies found that miR-574 negatively regulated Qki6/7 to increase the proliferation, migration and invasion of colorectal cancer [27], while Wang and colleagues proved that miR-574 suppressed lung metastasis of triple-negative breast cancer by targeting ZEB1 [28]. In the reproductive system, Pan and colleagues demonstrated that miR-574 negatively impacts oocyte quality and meiotic progression by targeting HAS2 during in vitro maturation (IVM) [22]; Zhang and colleagues found that miR-574 was associated with testis development and reproduction by targeting AURKA in white yak [21]. In the present study, we found an important role of miR-574 in sperm function by modulating mitochondrial function and reducing cellular ATP production through directly targeting mt-ND5. Mt-ND5 is an essential subunit of the mitochondrial respiratory chain I, which participates in establishing the proton gradient across the mitochondrial membrane that can be employed by the ATP synthase to drive ATP synthesis [29]. Recent reports have indicated that a mutation in mt-ND5 affects the respiratory complex and accounts for the ROS-dependent DNA damage response [30]. Our group recently found that Transplantation of the high-fat diet (HFD) gut microbes into the normal-diet (ND)-maintained (HFD-FMT) mice resulted in a significant decrease in spermatogenesis and sperm motility, and mt-ND5 was significantly decreased in the testes of HFD-FMT mice, suggesting an important role of mt-ND5 in testicular mitochondrial functions [31].

In our study, we confirmed the increase of miR-574 in the sperm of two aging mouse models and observed a negative relationship between sperm motility and miR-574. However, no such trend was observed in the clinical semen samples, suggesting that confounding factors other than age affected the detection. The cutoff for advanced paternal age used here is over 40 years old at the time of conception [32], and further study should be conducted with more stratification by age and should be performed on extensive semen samples in addition to the men attending infertility clinics. Herein, we proved the important role of miR-574 on sperm function in aging males, but the knockout or knock-in mice should be established to test the hypothesis in further investigations. Moreover, we observed that the embryos microinjected with miR-574 mimic showed a drop trend of embryonic development, with no statistical significance. Further study is required to examine the effect of pools of multiple miRNAs on early embryonic development.

In summary, our study delineates a miR-574-ND5 module that regulates mitochondrial function and ATP generation with respect to functional implications in the sperm of aging males. This shows that miR-574 plays important roles in sperm function and the regulatory signaling might offer a fresh view to comprehend the aging process in sperm.

## MATERIALS AND METHODS

### Animals, cell lines, and clinical sample collection

6-8 weeks old male C57BL/6 mice were purchased from Beijing Vital River Laboratory Animal Technology Co., Ltd. (Nanjing, China), housed on a 12 h light:12 h dark cycle at 22 ± 2 °C with free access to food and water. The procedures of animal experiments were executed according to the NIH guide for the care and use of laboratory animals, and were approved by the Ethics Committee of the Nanjing Jinling Hospital. Two aging mouse models were established as described previously [33–35]. In the D-gal-induced aging mouse model, D-gal (Sigma-Aldrich) (120 mg/kg/day) was injected subcutaneously into the mice daily for 42 days, and saline was given at the same volume subcutaneously in the control group. In the natural aging model, aged mice were raised routinely for more than 12 months as the Old group, and 6-8 weeks mice were as the Young group. Each group contained more than five mice at the end point of detection.

GC-2 (ATCC catalog number CRL-2196) and HEK293 cell lines were purchased from ATCC and cultured in high glucose Dulbecco’s modified Eagle’s medium (DMEM) supplemented with 10% fetal bovine serum (FBS) under 5% CO_2_ at 37 °C.

All clinical samples were collected according to protocols approved by the Medical Ethics Committee of Nanjing University, Jinling Hospital. All patients signed informed consent for the collection and use of their samples in this study. Those with a history of cryptorchidism, vascular trauma, orchitis, obstruction of the vas deferens, vasectomy, abnormalities in chromosome number or microdeletions of the azoospermia factor region on the Y chromosome were excluded from the study. Semen specimens were collected by masturbation after a period of 2–7 days of sexual abstinence and were kept to liquefy at 37 °C for 30 min. After liquefaction, semen parameters were analyzed using a computer-aided sperm analysis (CASA) system (CFT- 9201; Jiangsu Rich Life Science Instrument Co., Ltd., Nanjing, China). Sperm were obtained by centrifuging semen samples at room temperature at 2000 rpm for 5 min, then washed with PBS for 3 times and stored at -80 °C until use.

### Quantitative real-time PCR

Total RNA was isolated from sperm or GC-2 cells using a Total RNA Isolation Kit (BEI-BEI Biotech, Zhengzhou, China). For miRNA detection, complementary DNA (cDNA) was synthetized from total RNA using miRNA-specific primers (Ribobio). For mRNA detection, cDNA was synthetized from total RNA using PrimeScript^®^ RT Master Mix (Takara) reverse transcriptase according to the manufacturer’s instructions. cDNA was quantified by RT-qPCR using a Roche Light Cycler 96 Real-time PCR system (Roche Diagnostics, Basel, Switzerland). Real-time PCRs were performed in triplicate. β-Actin and U6 were used as endogenous controls for mRNA and miRNAs, respectively. Relative expression was calculated using the comparative ∆∆Ct method. The primers for miRNA real-time PCR were purchased from Ribobio Company (Guangzhou, China). The other primer sequences are presented in Supplementary Table 1.

### Western blotting

Western blotting was performed as described previously [36]. Proteins were harvested as indicated in figure legends, separated by sodium dodecyl sulfate polyacrylamide gel electrophoresis (SDS-PAGE) and transferred onto PVDF membranes. Dilution of 1:1000 for the anti-mt-ND5 antibody (55410-1-AP; Proteintech) and 1:5000 for the anti-β-Actin antibody (Proteintech) were used for primary antibody incubation. The blots were then incubated with HRP-conjugated secondary antibodies for 1 h at room temperature, prior to being processed using an ECL Plus Western Blotting Detection System. β-Actin was used as a loading control for western blots.

### Transient transfection

The miR-574 mimic and inhibitor were purchased from Ribobio Company (Guangzhou, China). Small interfering RNAs (siRNAs) directed against mt-ND5 were designed and synthesized by GenePharma Company (Shanghai, China). Transfection was performed with siRNA or miR-574 mimic/inhibitor and their respective negative control RNAs, using a Lipofectamine 2000 kit (Invitrogen, CA) according to the manufacturer’s instructions. Cells were harvested 24 h after transfection and used as needed.

### Immunofluorescence

GC2 cells were transfected with miR-574 mimics or inhibitor for 24 h, fixed with 4% paraformaldehyde for 30 minutes, permeabilized with 0.1% Triton X-100 for 10 minutes, blocked in 1% BSA-supplemented PBS, and then incubated overnight at 4 °C with anti-8-OHdG antibody (Abcam). The following day the samples were washed with PBS for 5 min three times and incubated with anti-rabbit IgG H&L secondary antibody for 1 h in the dark. DAPI was added to each dish and incubated for 10 min in the dark at room temperature. The intensity of 8-OHdG was visualized by a fluorescence microscope.

### Measurements of reactive oxygen species

A ROS Detection Kit (Jiancheng Bioengineering Institute, Nanjing, China) was used to measure the generation of reactive oxygen species (ROS) in GC2 cells. GC2 cells were transfected with miR-574 mimics or inhibitor for 24 h and then loaded with 1 μM 2,7-dichlorofuorescin diacetate (DCFH-DA) for 0.5 h. The procedure was conducted at 37 °C. The cells were subsequently rinsed three times with PBS, and the images were captured with a fluorescence microscope.

### ATP measurements

GC2 cells were collected, washed and lysed with the lysis buffer provided by an ATP Assay kit (Beyotime) after transfection with miR-574 mimics or inhibitor for 24 h. The ATP was detected according to the manufacturer’s instructions. The protein level of the lysate was measured with an Enhanced BCA Protein Assay Kit (Beyotime). The relative ATP level was calculated according to the following formula: relative ATP level= ATP value/protein value. All experiments were performed in three replicates.

### Flow cytometry detection of mitochondrial membrane potential

Flow cytometry was used to analyze mitochondrial membrane potential (MMP). Briefly, GC2 cells were harvested after transfection of miR-574 mimics or inhibitors for 24 h, and then stained with JC-1 according to the manufacturer’s instructions. The mitochondrial membrane potential detection kit (Beyotime) was used for cell staining. The stained cells were detected by a FACS Caliber (Becton Dickinson, Mountain View, NJ, USA) and analyzed using Modfit LT software (Becton Dickinson). All experiments were performed in three replicates.

### Histology and electron microscopy

Testes from C57BL/6 mice were collected and fixed in Bouin’s fixative for >12 h followed by embedding in paraffin. Paraffin sections (5-μm thickness) were further processed for eosin solution staining followed by hematoxylin counterstaining.

For electron microscopy observation, the freshly isolated testes were immersed in 2.5% glutaraldehyde for 12 h, and post-fixed in 1% osmium tetroxide for 1 h. After dehydration and embedding, the ultrathin sections were prepared and mounted on copper girds, double stained with uranyl acetate and citrate and examined using a HT7700 TEM (Hitachi, Japan).

### Computer-assisted sperm analysis

Epididymal sperm were prepared by computer-assisted semen analysis as described previously. Briefly, the epididymis was dissected, and sperm inside were squeezed out with forceps. After incubation in human tubal fluid medium (500 μl per epididymis) at 37 °C for 30 min, sperm were subjected to motility analyses using a Sperm Quality Analyzer. For each measurement, a suspension of spermatozoa was loaded into a microchamber slide with 100 μm depth. 300 spermatozoa were analyzed using the standard setting.

### Mitochondrial isolation

GC2 cells were collected, washed and homogenized with mitochondria isolate buffer from a Mitochondria Isolate kit (Beyotime). The sample was centrifuged at 1000 g for 10 min at 4 °C. Pellets were discarded and then centrifuged at 3500 g for 10 min at 4 °C to obtain the mitochondrial fraction and cytoplasmic fraction according to the manufacturer’s instructions. The samples were stored at −80 °C until the determination of the miRNA.

### Luciferase reporter assay

For miRNA target validation, the targeted sequence of mt-ND5 was PCR-amplified using mouse cDNA and cloned into the *XhoI-NotI* site downstream of the pmiR-RB-Report^TM^ vector (Ribobio), A construct containing mutated miRNA binding sites (seed sequence) in mt-ND5 was generated using the QuikChange site-directed mutagenesis kit (Stratagene). HEK293 cells were cultured in 24-well plates in DMEM supplemented with 10% FBS at 37 °C, and then co-transfected with wild type or mutated construct (200 ng/well) and miR-574 mimics (20 nM) using Lipofectamine 2000 reagent. At 24 h post-transfection, cell lysates were analyzed for luciferase activity using the Dual-Glo luciferase assay kit (Promega) according to the manufacturer’s protocol. Data were normalized against the Renilla luciferase values.

### RNA immunoprecipitation

GC2 cells were transfected with miR-574 mimics for 48 h, and RNA immunoprecipitation (RIP) experiments were performed using the Magna RIP™ RNA-Binding Protein Immunoprecipitation Kit (Millipore, USA) according to the manufacturer’s instructions. To demonstrate that the detected signals were from the RNA that was specifically bound, total RNA (input controls) and corresponding species IgG controls were performed simultaneously. Immunoprecipitations of AGO2 were performed using an anti-AGO2 antibody (Abcam) overnight at 4 °C. RNAs was extracted by phenol:chloroform:isoamyl alcohol method and subjected to qRT-PCR.

### Embryos collection, culture and miRNA microinjection

6-8-weeks-old female C57BL/6 mice received an injection of hCG 2 d after PMSG priming and were then mated overnight with 6-8-week-old males of proven fertility. Zygotes were obtained by flushing the oviducts 20 h after the hCG injection. Embryos were cultured in M2 medium under mineral oil at 37 °C in a 5% CO_2_, 5% O_2_ and 90% N_2_ incubator.

Microinjection of miR-574 mimics was used to overexpress miR-574 with a Narishige microinjector. For overexpression experiments, miR-574 mimics were diluted with water to give a stock concentration of 30 ng/μL, and then a 2.5 picoliter solution was injected into embryos. miR-NC was injected as a control.

### Statistical analysis

Results are recorded as means±SD for at least three independent experiments. The Student’s *t* test was used for continuous variables. The χ^2^ test was used to examine the relationships between miR-574 injection and embryo development. For statistical correlation, Pearson’s correlation coefficient was used according to requirement. Statistical analyses were performed using the SPSS software package (version 16.0; IBM SPSS, Chicago, IL). A P-value< 0.05 was considered statistically significant.

## Supplementary Material

Supplementary Figures

Supplementary Table 1
