# Supplementary material for: Mitochondria-related miR-574 reduces sperm ATP by targeting ND5 in aging males
Source: Aging (Albany NY). 2020 May 7;12(9):8321–38. doi: 10.18632/aging.103141 (PMC7244036; doi:10.18632/aging.103141)
Supplement: Supplementary Figures [file aging-12-103141-s002..pdf]

SUPPLEMENTARY FIGURES

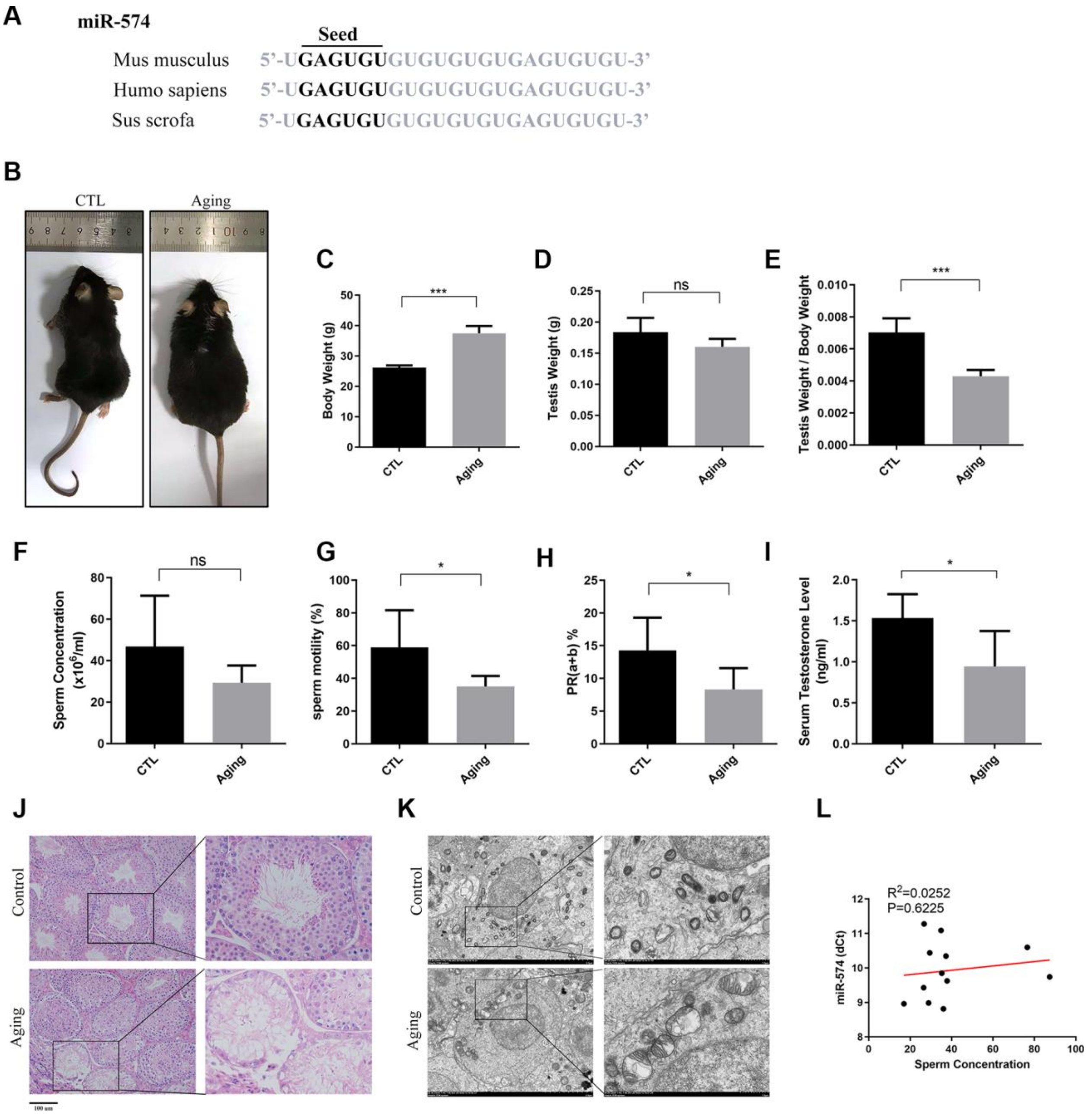

Supplementary Figure 1. Establishment of natural aging mouse model. (**A**) MiR-574 is highly conserved in multiple species. (**B**) The older-age mice presented thinning of hair, hypoactivity and other characteristics of aging. (**C**–**E**) The body weight, testicular weight and testicular organ index of the natural aging mouse model. (**F**–**I**) The sperm concentration, sperm motility, progressive motility and serum testosterone of the natural aging mouse model. (**J**–**K**) H&E staining and electron microscopy observations in the testes of the natural aging mouse model. (**L**) Scatter plot of sperm concentration and the miR-574 expression in the sperm of the natural aging mouse model.

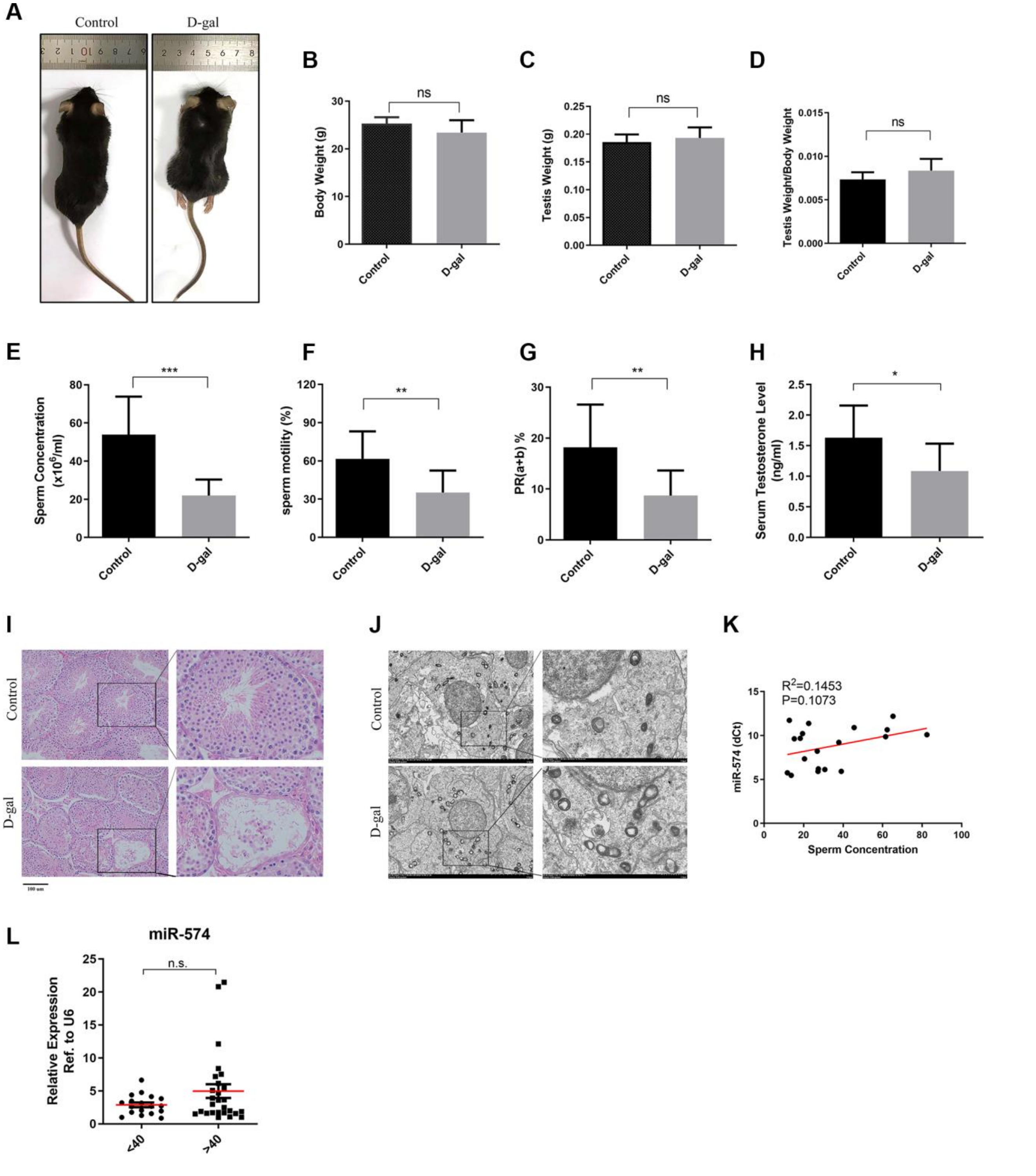

Supplementary Figure 2. Establishment of the D-gal-induced aging mouse model. (**A**) The D-gal-treated mice presented few characteristics of aging in appearance. (**B**–**D**) The body weight, testicular weight and testicular organ index of the D-gal-treated mouse model. (**E**–**H**) The sperm concentration, sperm motility, progressive motility and serum testosterone of the D-gal-treated mouse model. (**I**–**J**) H&E staining and electron microscopy observations in the testes of the D-gal-treated mouse model. (**K**) Scatter plot of sperm concentration and the miR-574 expression in the D-gal-treated mouse model. (**L**) miR-574 was detected in the sperm of males less than 40 years old (n=18) and more than 40 years old (n=28).

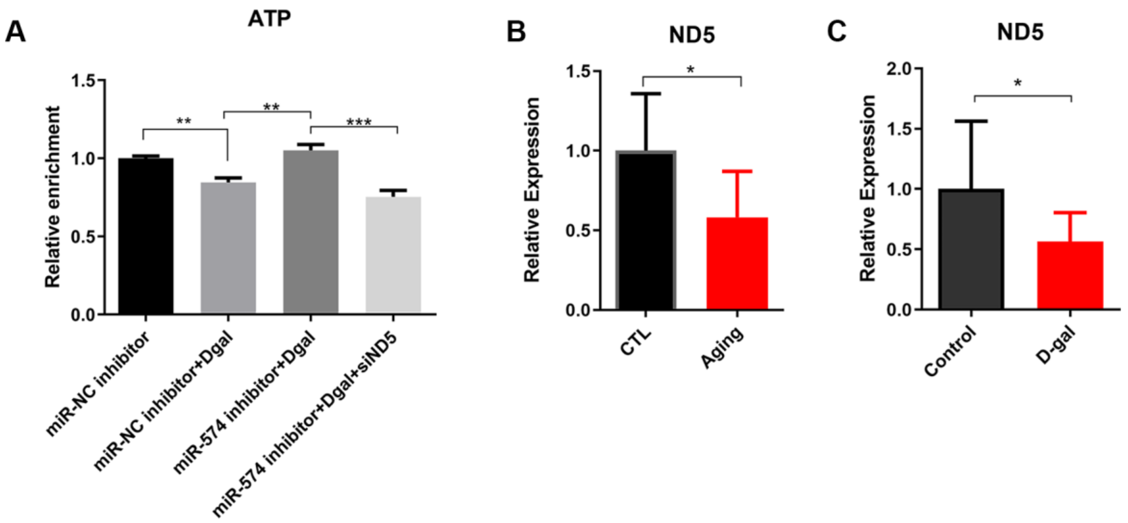

Supplementary Figure 3. mt-ND5 depletion alleviated the increase effects of miR-574 inhibitor on ATP levels. (**A**) The ATP levels was detected in different groups as shown. (**B**–**C**) The expression of mt-ND5 was detected in the sperm of the natural aging mouse model and D-gal-induced aging mouse model. Data are shown as mean±S.D.

A

Preimplantation development of embryos with or without miR-574 supplementation

Injected content	No. 2PN	No. 2-cell (% of 2-cell / 2PN)	No. 4-cell (% of 4-cell / 2-cell)	No. Blastocyst (% of Blastocyst / 2-cell)
miR-NC	81	74 (91.36)	71 (95.95)	57 (77.03)
miR-574	120	107 (89.17)	97 (90.65)	70 (65.42)

Note: 2PN, 2 pronuclei;

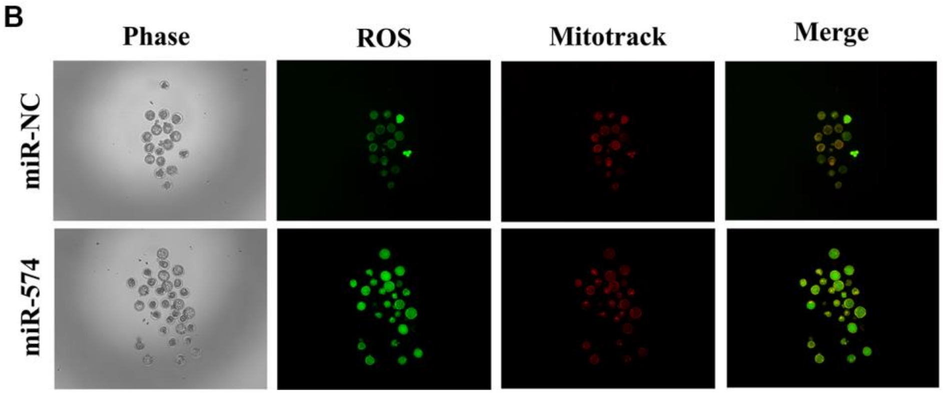

Supplementary Figure 4. Effects of miR-574 on early embryonic development. (**A**) The preimplantation development of embryos with or without miR-574 supplementation. (**B**) The ROS (green) and MitoTracker (red) were detected in the embryos with or without miR-574 supplementation.
