# Supplementary material for: Mitochondria-related miR-574 reduces sperm ATP by targeting ND5 in aging males
Source: Aging (Albany NY). 2020 May 7;12(9):8321–38. doi: 10.18632/aging.103141 (PMC7244036; doi:10.18632/aging.103141)
Supplement: Supplementary Table 1 [file aging-12-103141-s001..pdf]

SUPPLEMENTARY TABLE

Supplementary Table 1. Oligonucleotide sequences used in this study.

Gene description	Forward	Reverse
β-actin-q	5′-AGCCATGTACGTAGCCATCC-3′	5′-CTCTCAGCTGTGGTGGTGAA-3′
mt-ND5-q	5′-TCTCTACATCAAGCCAACT-3′	5′-GATTGAGCCAGAGCATAT-3′
GAPDH-q	5′-AGGTCGGTGTGAACGGATTTG-3′	5′-TGTAGACCATGTAGTTGAGGTCA-3′
mt-Cytb-q	5′-AACATACGAAAAACACACCCATT-3′	5′-AGTGTATGGCTAAGAAAAGACCTG-3′
mt-ND5-siRNA1	5′-CCUACAAGCAAUCCUCUAUTT-3′	5′-AUAGAGGAUUGCUUGUAGGTT-3′
mt-ND5-siRNA2	5′-GGAAGCAUCUUUGCAGGAUTT-3′	5′-AUCCUGCAAAGAUGCUUCCTT-3′
mt-ND5-RIP-1	5′-TATAACCGCATCGGAGAC-3′	5′-TGGTAGTCATGGGTGGAG-3′
mt-ND5-RIP-2	5′-GCCTCCACCCATGACTACCA-3′	5′-AGGGCTCCGAGGCAAAGTA-3′
mt-ND5-RIP-3	5′-TCTCTACATCAAGCCAACT-3′	5′-GATTGAGCCAGAGCATAT-3′
mt-ND5-WT	5′-GCGCTCGAGTACCATGCTTATCCTCACCTCAG-3′	5′-AATGCGGCCGCCTAACCAGATCAAGTCTAGGAGAG-3′
mt-ND5-Mut	5′-GCACTACTTGTGAGAAGTACAATAGTAGTTGC-3′	5′-TTGTACTTCTCACAAGTAGTGCTGAAACTGGT-3′
